# Genome-wide mRNA profiling identifies the NRF2-regulated lymphocyte oxidative stress status in patients with silicosis

**DOI:** 10.1186/s12995-021-00332-0

**Published:** 2021-09-13

**Authors:** Yingzheng Zhao, Guangcui Xu, Haibin Li, Meiyu Chang, Cheng Xiong, Yingjun Tao, Yi Guan, Yuchun Li, Sanqiao Yao

**Affiliations:** 1 School of Public Health, North China University of Science and Technology, Tangshan, Hebei Province 063009 People’s Republic of China; 2 School of Public Health, Xinxiang Medical University, Xinxiang, Henan Province 453003 People’s Republic of China

**Keywords:** Silicosis, Nuclear factor-erythroid 2-related factor 2, Fibrosis, GSEA-analysis, Peripheral blood mononuclear cells

## Abstract

**Background:**

The immunomodulatory abnormalities of silicosis are related to the lymphocyte oxidative stress state. The potential effect of antioxidant therapy on silicosis may depend on the variation in nuclear factor erythroid 2-related factor 2 (NRF2)-regulated antioxidant genes in peripheral blood mononuclear cells (PBMCs). As NRF2 is a redox-sensitive transcription factor, its possible roles and underlying mechanism in the treatment of silicosis need to be clarified.

**Methods:**

Ninety-two male patients with silicosis and 87 male healthy volunteers were randomly selected. PBMCs were isolated from fresh blood from patients with silicosis and healthy controls. The lymphocyte oxidative stress state was investigated by evaluating NRF2 expression and NRF2-dependent antioxidative genes in PBMCs from patients with silicosis. Key differentially expressed genes (DEGs) and signaling pathways were identified utilizing RNA sequencing (RNA-Seq) and bioinformatics technology. Gene set enrichment analysis was used to identify the differences in NRF2 signaling networks between patients with silicosis and healthy controls.

**Results:**

The number of monocytes was significantly higher in patients with silicosis than that of healthy controls. Furthermore, RNA-Seq findings were confirmed using quantitative polymerase chain reaction and revealed that NRF2-regulated DEGs were associated with glutathione metabolism, transforming growth factor-β, and the extracellular matrix receptor interaction signaling pathway in PBMCs from patients with silicosis. The top 10 hub genes were identified by PPI analysis: SMAD2, MAPK3, THBS1, SMAD3, ITGB3, integrin alpha-V (ITGAV), von Willebrand factor (VWF), BMP4, CD44, and SMAD7.

**Conclusions:**

These findings suggest that NRF2 signaling regulates the lymphocyte oxidative stress state and may contribute to fibrogenic responses in human PBMCs. Therefore, NRF2 might serve as a novel preventive and therapeutic candidate for silicosis.

## Introduction

Silicosis is a pulmonary interstitial fibrosis disease caused by the exposure to crystalline silica dust. This disease is a progressive process characterized by lung inflammation during the early phase and triggering pulmonary fibrosis with subsequent impairment of lung function [1, 2]. Unfortunately, no effective treatments are available, except the possibility of a lung transplant in a small minority of patients [3]. At present, a large number of employees are exposed to high concentrations of dust in a booming mining industry in China. The number of new pneumoconiosis reported per year reached more than 20,000 in China, almost half of which was silicosis [4].

Silicosis is characterized not only by its direct fibrotic effect on lung tissue but by immunomodulatory abnormalities, such as the appearance of complications of autoimmune diseases and autoantibodies in silica-exposed populations [5–7]. Peripheral blood mononuclear cells (PBMCs) are collected from the peripheral or circulating blood and possess a single, round nucleus. PBMCs are the major immune cells in the human body and provide selective responses to the immune system. The roles of these blood cells are to adapt to intruders and fight infection. PBMCs mainly comprise T-lymphocytes, B-lymphocytes, and natural killer (NK) cells and are also a rich source of monocytes, such as macrophages and dendritic cells [8]. It was reported that lymphocytes account for 70–95% in PBMCs [9, 10]. PBMCs play a crucial role in maintaining and controlling the immunomodulatory process in silicosis pathogenesis [11]. It has been reported that the gene associated with apoptosis was significantly overexpressed in PBMCs derived from patients with silicosis and the dysregulation of the genes in silicosis cases could cause the appearance of autoantibodies and acquisition of autoimmune diseases sequentially [12, 13]. Animal experiments demonstrated that silica exposure induces migration of dendritic cells from the peripheral blood into the alveoli in rats [14]. Taken together, PBMCs are associated with the pathogenesis or development of silicosis.

Oxidative stress occurs due to a persistent imbalance of redox homeostasis induced by overproduction of reactive oxygen species (ROS) in silica-exposed populations. When ROS production exceeds antioxidant capacity, oxidative stress may have harmful effects on the structure and function of biological tissues. It is essential for oxidative damage to occur in the development of silicosis [15–17]. It was showed that the antioxidant N-acetylcysteine alleviates lung fibrosis induced by silica in rats by downregulating ROS and interfering with apoptosis signaling in our previous study [18]. Antioxidant treatments have been shown to be effective for silicosis in animal experiments [19–21]. However, antioxidant therapy has produced conflicting results in clinical studies of the treatment of pulmonary fibrosis and other chronic diseases [22, 23]. The failure of clinical trials may be due to the lack of understanding of the role of ROS in the development of silicosis. Indeed, clinical effects of antioxidant therapy on silicosis depend on the redox microenvironment, which changes spatially and temporally in different cell types and in different subcellular compartments [24]. Thus, the individual’s difference in redox microenvironment potentially depends on variation of their antioxidant genes. We hypothesize that the endogenous antioxidant levels in the subjects might have greatly affected the results of the clinical trials.

Antioxidant defense systems to combat ROS from tissues oxidative damage include enzymatic antioxidants and nonenzymatic antioxidants. Enzymatic antioxidants comprise superoxide dismutases (SODs), catalase (CAT), glutathione peroxidases (GPXs), glutathione reductase (GR), and aldehyde dehydrogenases (ALDH). Glutathione (GSH), coenzyme Q10, ascorbic acid (vitamin C) and α-tocopherol (vitamin E) represent nonenzymatic antioxidants, which influence each other and have their own unique roles [25]. The GPx/GR antioxidant system is related to other antioxidant systems, including the SOD/CAT system. GSH modulates the neutralization of free radicals by vitamin C (ascorbic acid) and vitamin E [26]. Occupational silica exposure leads to the alteration of SOD and CAT activities in the antioxidant defense system [27]. Oxidative stress status as demonstrated by the level of antioxidant biomolecules could provide an early warning signal in diseases related to oxidative damage such as cancer, cataract, and heart disease [28].

Nuclear factor erythroid 2-related factor 2 (NRF2) is a redox-sensitive transcription factor and plays a critical role in maintaining redox homeostasis by regulating the expression of antioxidant defense enzymes [29]. Levels of NRF2 in PBMCs increase immediately after ozone/oxygen exposure [30]. In a human intervention study, antioxidant modulated the NRF2- related gene expression in PBMCs [31]. Our previous research shows that NRF2 is involved in mediating the development of silicosis in animal experiments [32]. Although studies in animals have preliminarily revealed that the pathogenic process of silicosis associated with oxidative stress is regulated by NRF2, the integrated mechanism requires further exploration. Advances in molecular biology and genomics have enabled further exploration as high-throughput sequencing technology has been widely used in disease diagnosis and prognosis. However, gene expression profile changes have rarely been discussed in PBMCs of patients with silicosis.

In the present study, genome-wide mRNA profiling was used to identify the lymphocyte oxidative stress state regulated by NRF2 in patients with silicosis, and to investigate whether there was an association between lymphocyte oxidative stress state and the pathogenesis of silicosis. RNA sequencing (RNA-Seq) technology and bioinformatics technology were comprehensively integrated and subsequently identified key differentially expressed genes (DEGs) and signaling pathways. The role of DEGs in silicosis was explored from three aspects, including cellular components, molecular function, and biological process, which were also used to mine potential pathogenic genes and biomarkers. Furthermore, the clinical significance of identified genes was verified using clinical samples. These data provide novel information and further understanding of the mechanism underlying oxidative stress in PBMCs from patients with silicosis.

## Materials and methods

### Study design and participants

A total of 92 male patients with silicosis (the range of age: 40–65 years) and 87 male healthy volunteers (the range of age: 40–65 years) were randomly selected from an institute of occupational disease prevention and control from Henan Province in the study. The healthy controls group had no dust exposure in the past. The group of patients with silicosis were strictly selected according to the standard diagnoses of pneumoconiosis (GBZ70–2015), and the patients were diagnosed as silicosis stage I. All subjects had no clinical symptoms of autoimmune disease, including Raynaud’s phenomenon, sclerotic skin, arthralgia or facial erythema, or malignant tumors. Additionally, all subjects were negative for pneumonia, active pulmonary tuberculosis, pulmonary heart disease, infectious diseases and other lung related diseases. All subjects confirmed that they understood the experimental procedure and provided written informed consent. The study was approved by the Ethics Committee of the Xinxiang Medical University (protocol number XYLL-2017086, approved 3 March 2017).

In this study, information was collected using selected survey questionnaires by trained medical personnel. The survey mainly included information related to age, smoking status, basic health status, and family history. Physical examinations included blood pressure, weight, and height measurements to determine body mass and body mass index (BMI). Levels of PBMCs in every subject were detected by an automated hematology analyzer (Cell-Dyn Sapphire, Abbott, USA). Pulmonary function evaluation was performed using a portable handheld spirometer (Drägerwerk AG, Lűbeck, Germany). The general specifications for the performance, as well as interpretation, of the pulmonary function test were followed [33, 34]. The pulmonary function indices documented were the forced vital capacity (FVC) and forced expiratory volume in 1 second (FEV1). All readings were in units of liters and percentage predicted values. The transforming growth factor (TGF)-β1 content was detected in accordance with manufacturer instructions by ELISA. In addition, three patients with silicosis and three healthy donors were randomly selected for the detection of genome-wide mRNA profiling. Thirty-six participants were respectively selected from the remaining healthy and patient samples with random and double-blindness method for mRNA analysis, immunofluorescence and western blot analysis.

### PBMC isolation

PBMCs were isolated from 10 ml of fresh blood from patients with silicosis and healthy controls within 30 min after collection using the Ficoll-Paque (GE Healthcare Bio-Sciences, Pittsburgh, PA, USA) density centrifugation method. Briefly, blood was diluted 1:1 with RPMI-1640 medium at room temperature (Solarbio, Beijing, China), underlaid with 10 ml Ficoll-Paque, and then centrifuged (1000×*g*, 10 min, 20 °C) with acceleration set to six and deceleration set to zero in a Heraeus Multifuge X3R (Thermo Fisher Scientific, Langenselbold, Germany). Separated PBMCs were carefully collected and transferred to a 50 ml conical tube, which was brought up to 50 ml with RPMI-1640 medium, and then washed three times with RPMI-1640 medium (100×*g*, 10 min, 4 °C) with acceleration set to nine and deceleration set to six. The supernatant was removed and the pellet was resuspended in 1.0 ml RPMI-1640 medium for total RNA extraction, immunofluorescence assays or western blotting. PBMCs were enumerated and assessed for viability using trypan blue. Samples were stored at − 80 °C before use.

### Expression of NRF2 in PBMCs of patients with silicosis using immunofluorescence

After PBMCs were isolated from peripheral blood, the expression of NRF2 in PBMCs of patients with silicosis was determined using a fluorescent immunocytochemistry assay. PBMCs were diluted to 2 × 10^6^/ml with phosphate-buffered saline (PBS). A total of 300 μl of diluted suspension was spread evenly over the slide and allowed to air-dry. Then, PBMCs were fixed with 4% paraformaldehyde for 20 min at room temperature and washed three times with PBS. Sections were incubated with primary antibodies to NRF2 (Abcam, 1:100) overnight at 4 °C and then incubated with Cy3-conjugated secondary antibodies (1:200) at room temperature for 50 min in the dark. Nuclei were labeled using 4′, 6-diamidine-2′-phenylindole dihydrochloride and the results observed under a confocal laser scanning microscope (TCS STED-CW, Leica Microsystems, Mannheim, Germany). Sixteen areas per sample covering the whole spectrum of blood smear, were evaluated in a blinded fashion. Immunofluorescence images were analyzed using Image J Software. Finally, fluorescent intensity of NRF2 staining was assessed by a pathologist blinded to study groups.

### Expression of NRF2 and NRF2 regulated proteins in PBMCs of patients with silicosis using western blotting

Total proteins were extracted from PBMCs isolated from peripheral blood. Protein concentration was quantified using a BCA assay kit (Thermo, USA). Equal amounts of total protein were separated by 10% sodium dodecyl sulfatepolyacrylamide gel electrophoresis and then transferred onto a polyvinylidene fluoride membrane (Millipore). After blocked for 2 h in 5% nonfat milk, they were incubated with the primary antibodies: NRF2 (1:1000) (Affinity Biosciences, USA), GCLM (1:2000) (Abcam, USA), KEAP-1(1:2000, Cell Signaling, Boston, MA, USA), anti-GAPDH antibodies (1:5000, Proteintech, USA) overnight at 4 °C. The membranes were washed for 3 times in 1% Tris-buffered saline with Tween (TBST) containing 0.01% Tween 20 and incubated with horseradish peroxidase conjugated secondary antibodies (1:3000, Affinity Biosciences, USA) for 1.0 h at room temperature. After washing membranes, immunoreactive bands were visualized using a Fluor Chem R imaging analysis system (Protein Simple, California, USA). Finally, the density of proteins in each band were analyzed using Image J Software. All western blot analyses were performed at least three times.

### Total RNA extraction, library construction, and sequencing

Total cellular RNA was extracted from PBMCs using the TRIzol reagent (Gibco BRL Life Technologies, Gaithersburg, MD, USA), according to the manufacturer’s instructions. Extracted total RNA was analyzed using the Agilent 2100 RNA 6000 Nano kit (Agilent Technologies, Missisauga, Ontariao, Canada) to confirm its quality and quantity. An mRNA library was constructed using the NEB Next Ultra RNA library prep kit (New England Biolabs, Ipswich, MA, USA). An Agilent 2100 (Agilent, Santa Clara, CA, USA) was used for library quality inspection and an Illumina HiSeq TM 4000 (Illumina, Santa Clara, CA, USA) was used for sequencing. mRNA sequencing was performed by Gene Denovo Biotechnology Co. (Guangzhou, China). To ensure data quality, the original data was filtered to reduce data noise before analysis.

### Genome-wide mRNA profiling

#### DEG analysis

Bioinformatics was used to reanalyzed the original data from the sequencing results. Principal component analysis was performed using the R statistical software package with normalized counts to investigate if samples from the same group clustered together. The R-based software package edgeR was used to determine whether there was a significant difference in gene expression levels between the two groups. Genes with a fold change ≥2 and *P* < 0.05 in a comparison were classified as significant DEGs. The gene ontology (GO) project provides a uniform way to describe the functions of gene products and enable analysis of genomic data with respect to three aspects including molecular function, cellular component, biological process. Functional analysis of DEGs was performed using the GO project (http://www.geneontology.org) [35]. KEGG is a database resource for understanding biological functions of the genes obtained by genome sequencing through intuitional graphics. Pathway enrichment analysis was performed to identify significant pathways involving DEGs using the KEGG database [36].

#### Gene set enrichment analysis (GSEA)

GSEA is a method to evaluate genomic data at the level of gene sets. It can detect subtle enrichment signals and avoid ignoring genes with insignificant differences. The biological functions of the key NRF2-driven genes were clarified using GSEA (http://software.broadinstitute.org/gsea/index.jsp) [37]. A nominal *P* < 0.001 and a normalized enrichment score ≥ 1.00 were chosen as the cutoff criteria.

#### Co-expression network analysis

To reveal functional associations between proteins, the STRING database was used to construct a PPI network [38]. In the PPI network, each node represents a protein and each edge (between two nodes) represents an interaction between these two proteins. Hub proteins were the nodes with a relatively large number of edges. Within the network analysis, the degree of association is an important factor to determine the relative importance of a gene. Different colors and sizes of nodes were employed to discriminate the degree of associations for one gene with the surrounding nodes. The co-expression networks were constructed using Cytoscape [39].

#### Quantitative real-time polymerase chain reaction (RT-qPCR) validation of responses in patients with silicosis

RT-qPCR analysis was performed as previously described [18]. Total RNA was extracted from PBMCs of healthy controls and patients with silicosis using the TRIzol reagent (Invitrogen, San Diego, CA, USA) and reverse-transcribed by the Maxima First Strand cDNA Synthesis Kit for RT-qPCR (Thermo Scientific, USA). The relative expression levels of mRNAs encoding *NRF2*, Kelch-like ECH-associated protein 1 (*KEAP1*), glutamate-cysteine ligase modifier subunit (*GCLM*), *TGF-β*, mothers against decapentaplegic homolog 3 (*SMAD3*), *SMAD2*, mitogen-activated protein kinase 3 (*MAPK3*), thrombospondin 1 (*THBS1*), integrin beta-3 (*ITBG3*), bone morphogenetic protein 4 (*BMP4*), CD44 molecule (Indian blood group) (*CD44*), and glyceraldehyde 3-phosphate dehydrogenase (*GAPDH*) were measured using a LightCycler® 96 (Roche Diagnostics, Mannheim, Germany). The genes were amplified using SYBR Green PCR SuperMix (SYBR High-Sensitivity qPCR SuperMix, Novoprotein, Shanghai, China) and 300 nM of each primer pair. Primers were designed using the Primer Design-online Software (Sangon Biotech, Shanghai, China) and synthesized by Sangon Biotech (Shanghai, China). Normalized gene expression levels are provided as the ratio between the mean value for the target gene and that for the reference gene (GAPDH) in each sample. All reactions were repeated three times. All RNA samples exhibited a 260/280 ratio>1.8. The primer sequences used are shown in Table 1.

**Table 1:** Gene name and GenBank accession number used in quantitative reverse transcriptase PCR

Gene	Accession No.	Gene name	Primer sequence
***NRF2***	NC_000002.12	nuclear factor, erythroid 2 like 2	*5′-ACGGTATGCAACAGGACATTGAGC-3′* *5′-TTGGCTTCTGGACTTGGAACCATG-3’*
***KEAP1***	NC_000019.10	kelch like ECH associated protein 1	*5’-ATTCAGCTGAGTGTTACTACCC-3′* *5′-CAGCATAGATACAGTTGTGCAG-3’*
***GCLM***	NC_000069.6	glutamate-cysteine ligase, modifier subunit	*5’-GGGCACAGGTAAAACCAAATAG-3′* *5′-TTTTCACAATGACCGAATACCG-3’*
***SMAD3***	NC_000015.10	SMAD family member 3	*5’-AGAGAGTAGAGACACCAGTTCT-3′* *5′-GAAGTTAGTGTTTTCGGGGATG-3’*
***SMAD2***	NC_000018.10	SMAD family member 2	*5’-CTCTTCTGGCTCAGTCTGTTAA-3′* *5′-AAGGAGTACTTGTTACCGTCTG-3’*
***MAPK3***	NC_000016.10	Mitogen-activated protein kinase 3	*5’-TCTGCTACTTCCTCTACCAGAT-3′* *5′-CAGGCCGAAATCACAAATCTTA-3’*
***THBS1***	NC_000015.10	thrombospondin 1	*5’-TTTGACATCTTTGAACTCACCG-3′* *5′-AGAAGGAGGAAACCCTTTTCTG-3’*
***ITBG3***	NC_000017.11	Integrin β-3	*5’-TCATCTGGAAACTCCTCATCAC-3′* *5′-GTAGACGTGGCCTCTTTATACA-3’*
***LTPB1***	NC_000002.12	latent transforming growth factor beta binding protein 1	*5’-GCGATGAGTTGAACAACCGGATGTC-3′* *5′-TCAAGGCGGTATTCATCGGAGTGC-3’*
***TGFβ***	NC_000019.10	transforming growth factor beta 1	*5’-TGAACCGGCCTTTCCTGCTTCTCATGG-3′* *5′-GCGGAAGTCAATGTAGAGCTGCCGC-3’*
***BMP4***	NC_000014.9	Bone morphogenetic protein 4	*5’-GGTGGGAAACTTTTGATGTGAG-3′* *5′-TTGAGGTAACGATCGGCTAATC-3’*
***CD44***	NC_000011.10	CD44 molecule	*5’-TCTGAATCAGATGGACACTCAC-3′* *5′-CATTGCCACTGTTGATCACTAG-3’*
***ALOX12***	NC_000017.11	arachidonate 12-lipoxygenase, 12S type	*5’-GATCCGAGGAGAGAAGCAATAC-3′* *5′-TGAGTGTTCAGCAAGTGATACT-3’*
***GAPDH***	NC_000012.12	glyceraldehyde-3-phosphate dehydrogenase	*5’-AAGAAGGTGGTGAAGCAGGC-3′* *5′-TCCACCACCCTGTTGCTGTA-3’*

#### Statistics

Routine test data were analyzed using SPSS 19.0 analysis software. Data are reported as the mean ± SD values. Group differences were calculated using a *t*-test and a Chi-squared test. The pearson’s correlation coefficients were calculated to test the relationship between the variables. RNA sequencing data between two groups were analyzed using the R statistical software package. *P* < 0.05 was considered to be significantly different.

## Results

### Clinical and cytology characteristics of patients with silicosis and healthy volunteers

A total of 92 male patients with silicosis and 87 male healthy volunteers participated in this study; among them, only 77 patients with silicosis and 62 healthy controls fully satisfied the enrollment criteria. Clinical and peripheral blood cytology characteristics of the subjects are described in Table 2. There were no statistical differences in age, BMI, blood pressure, and smoking status between the two groups. FVC and FEV1 for respiratory function were significantly lower in the group of patients with silicosis than that of healthy controls. The number of monocytes was significantly higher in patients with silicosis than that of healthy controls through cytology detection, indicating that monocytes may play an important role in cellular responses induced in patients with silicosis (Table 2). The clinical and cytology characteristics in the samples used for the biomolecular detection and Genome-wide mRNA profiling are similar to that of all silicosis patients and healthy controls (Table 2).

**Table 2:** Clinical and cytology characteristics of silicosis patients and healthy volunteers

Characteristics	Overall		Samples for detection of Nrf2-related molecular		Samples for RNA sequencing	
	Healthy Controls(*n* = 62)	Silicosis Patients(*n* = 77)	Healthy Controls(*n* = 36)	Silicosis Patients(*n* = 36)	Healthy Controls(*n* = 3)	Silicosis Patients(*n* = 3)
Age (years)	52.56 ± 6.18	51.55 ± 8.38	52.19 ± 6.49	54.31 ± 7.50	52.67 ± 9.07	53.67 ± 9.29
BMI (kg/m^2^)	25.01 ± 3.36	25.47 ± 3.15	25.43 ± 3.34	25.62 ± 2.78	22.79 ± 2.66	27.25 ± 2.93
Smokers/non-Smokers	32/45	28/38	16/20	15/21	0/3	0/3
SBP (mmHg)	126.13 ± 15.38	130.00 ± 14.60	123.47 ± 14.63	128.19 ± 9.19	130.00 ± 10.00	125.00 ± 5.00
DBP (mmHg)	84.84 ± 9.19	85.00 ± 9.42	84.03 ± 8.77	82.64 ± 6.38	85.00 ± 5.00	76.67 ± 5.77
Percentage of FVC (%)	91.05 ± 9.24	80.81 ± 23.54^**#**^	93.19 ± 6.68	79.97 ± 18.97^**#**^	89.33 ± 4.04	66.63 ± 7.51^**#**^
Percentage of FEV_1.0_ (%)	94.05 ± 7.67	84.35 ± 19.68^**#**^	90.72 ± 8.70	76.18 ± 23.59^**#**^	91.33 ± 5.51	67.07 ± 9.64*
WBC (×10^6^/L)	5.90 ± 1.35	6.04 ± 1.28	5.88 ± 1.37	5.85 ± 1.27	5.70 ± 1.05	6.59 ± 0.92
Neutrophils (× 10^6^/L)	3.70 ± 1.02	3.40 ± 1.16	3.51 ± 0.72	3.26 ± 0.87	3.77 ± 0.93	3.67 ± 0.79
Monocytes (×10^6^/L)	0.23 ± 0.09	0.47 ± 0.15^**#**^	0.23 ± 0.08	0.40 ± 0.10^**#**^	0.20 ± 0.10	0.38 ± 0.16*
Lymphocytes (×10^6^/L)	2.07 ± 0.65	2.03 ± 0.65	1.97 ± 0.63	1.99 ± 0.75	1.73 ± 0.32	2.06 ± 0.67
TGF-β(pg/mL)	31.26 ± 3.23	44.58 ± 3.42^**#**^	28.85 ± 3.22	47.59 ± 4.98*	25.20 ± 7.56	62.54 ± 4.89^**#**^

*Note*: Continuous variables are expressed as mean ± SD

*BMI* body mass index, *SBP* systolic blood pressure, *DBP* diastolic blood pressure, *FVC* forced vital capacity, *FEV1.0* forced expiratory volume in one second, *WBC* white blood cell

*P < 0.05 vs Healthy Controls; **#** *P* < 0.01 vs Healthy Controls. *P* values determined by *t*-test and Chi-squared test

### Expression of NRF2 and NRF2-dependent antioxidative genes in PBMCs from patients with silicosis

**Fig. 1:**
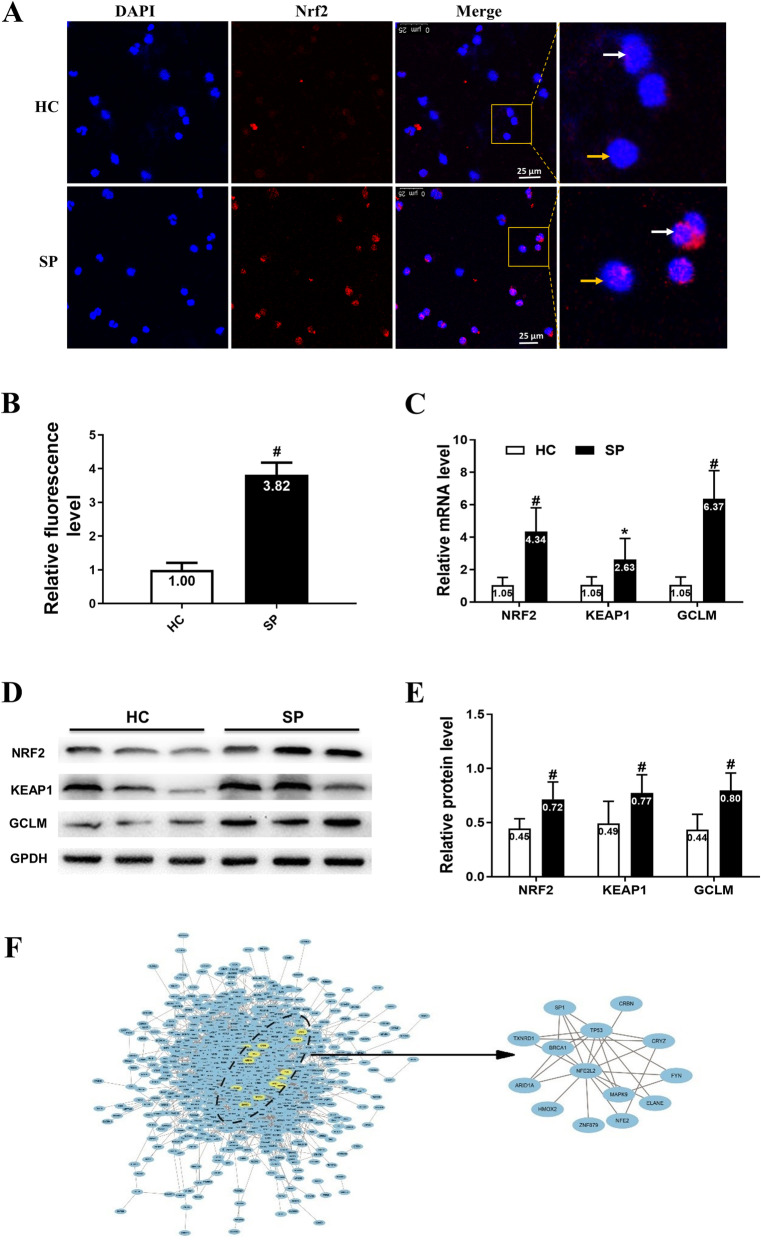
The expression of Nrf2 and Nrf2-dependent antioxidative genes in silicosis patients PBMCs. **A** The represent images of Nrf2 expression in PBMCs isolated from silicosis patients and healthy controls by confocal laser scanning microscope. **B** Semi-quantitative analysis of Nrf2 fluorescence levels in PBMC from the two groups. **C** The expression of Nrf2 and Nrf2-dependent antioxidative genes in silicosis patients PBMCs by real-time PCR. **D** Western blot analysis of Nrf2 and Nrf2-dependent proteins levels in silicosis patients PBMCs. **E** Protein levels of Nrf2 and Nrf2-dependent proteins were quantified using Image J Software. **F** DEGs related to NRF2 in the development of silicosis. Data are expressed as fold changes relative to the healthy control group. Horizontal line represents the mean fold change. **A** Yellow arrows: lymphocytes and White: monocytes. **B**, **C**, **E** Error bar indicates mean ± SD (n = 36 per group). * *P* < 0.05 vs. HC, **#** *P* < 0.01 vs. HC

To investigate the potential role of *NRF2* in PBMCs from patients with silicosis, the expression of *NRF2* was examined using a confocal laser scanning microscope. Notably, the expression of *NRF2* was significantly higher in patients with silicosis than in healthy controls, and it is showing a similar tendency in the lymphocytes and the monocytes (Fig. 1A). Semi-quantitative analysis revealed a significantly higher NRF2 fluorescence level in patients with silicosis compared to that in healthy controls (Fig. 1B). Moreover, RT-qPCR analysis showed that the expression of *NRF2*, *KEAP1* and *GCLM* mRNA in PBMCs from patients with silicosis was higher than that in healthy controls (Fig. 1C). Accordingly, the protein expression in PBMCs from patients with silicosis were measured by western boltting. The results of western bolt showed the protein expression of NRF2, KEAP1 and GCLM in PBMCs was higher in patients with silicosis than that of healthy controls (Fig. 1D and E). These results showed that the expression levels of *NRF2* and *NRF2*-dependent antioxidative genes increased in PBMCs from patients with silicosis. Network analysis of the DEGs from transcriptome sequencing found that the genes in the NRF2 module related to *NRF2* involved in the silicosis, which suggests that DEGs associated with NRF2 might affect the pathogenesis of silicosis (Fig. 1F).

### Identification of DEGs in the PBMCs of patients with silicosis

In total, six samples were included: three samples in the healthy control group and three samples in the group of patients with silicosis. Using commercial RNA preparation kits, total RNA was prepared from PBMC samples and the integrity number (RIN) value of each sample is greater than 8.9.

**Fig. 2:**
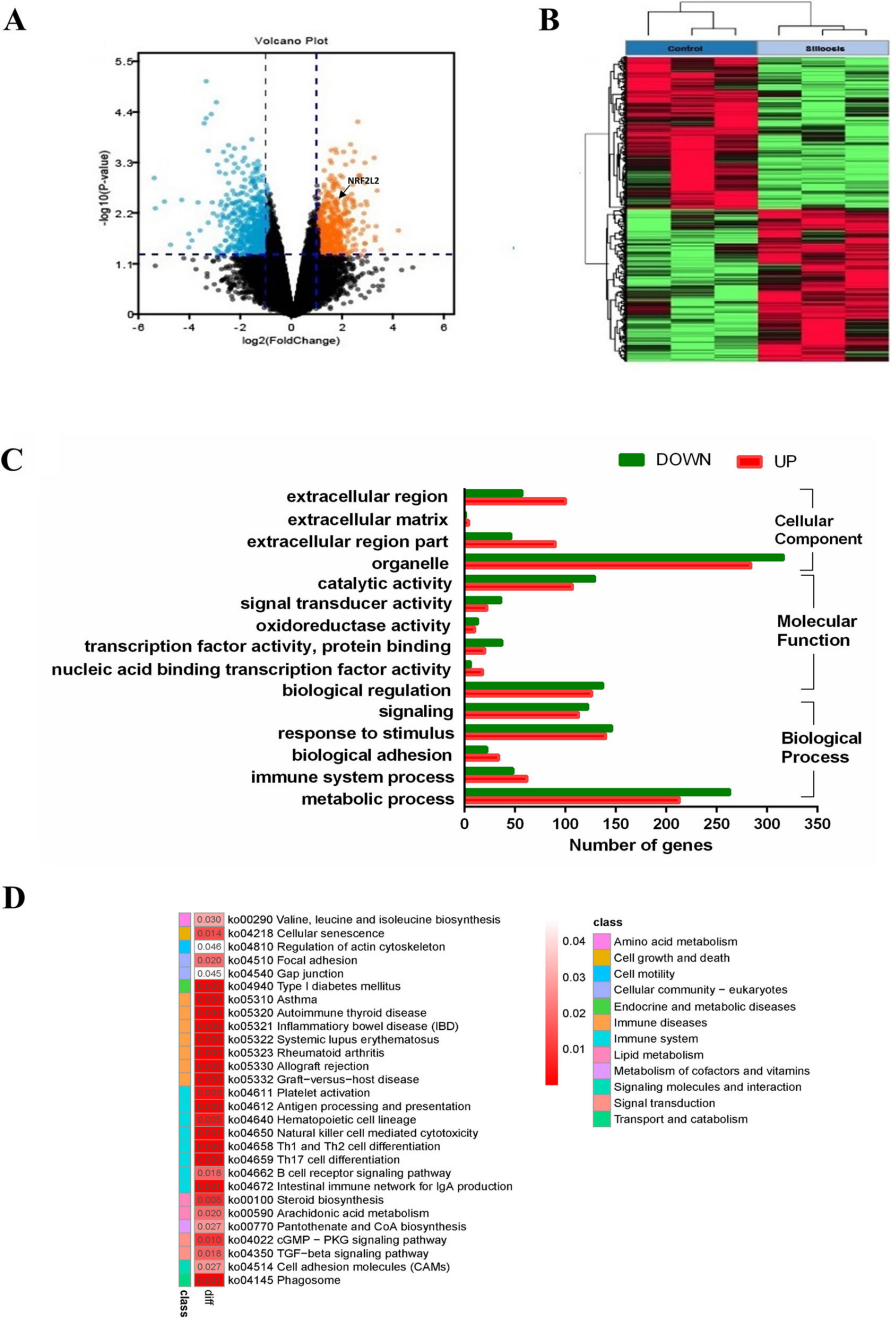
RNA-seq transcriptomic profiles and the differential expressed genes in the PBMC between HC and SP groups. **A** Volcano plot. Red dots represent upregulated DEGs, the blue dots represent downregulated DEGs, and black dots represent non-DEGs. DEGs, differentially expressed genes. **B** Heatmap of the top 500 genes between HC and SP groups. s**C** GO analysis of differentially expressed genes in the development of silicosis. **D** KEGG analysis of differentially expressed genes in the development of silicosis. (*n* = 3 per group)

To identify key pathways and genes in the silicosis, the differentially expressed mRNAs among healthy controls and patients with silicosis were analyzed using the Gene-e software. Three conditions were set for DEG screening: a false discovery rate < 20%, unfolded change > 2 and *P* < 0.05. A total of 1158 genes were identified after the analysis, of which, 475 were upregulated and 683 were downregulated (Fig. 2A and B).

### Key molecules and signaling pathways were screened through annotation of DEGs in the silicosis

GO analysis was performed to explore potential roles of differentially expressed mRNAs in the silicosis. GO analysis showed that the DEGs between patients with healthy controls and patients with silicosis were mainly involved in cellular component (including organelle, extracellular region, extracellular matrix, and extracellular region part), oxidation reduction (such as catalytic and oxidoreductase activity), transcription factor activity (such as transcription factor activity, protein binding, and nucleic acid binding transcription factor activity), regulating metabolic processes, immune system processes, response to stimulus transcription, regulating signal transduction, and biological adhesion (Fig. 2C).

KEGG pathway analysis showed that the DEGs between patients with silicosis and healthy controls were primarily enriched in pathways associated with regulating immune-related pathways (Graft-versus-host disease, Rheumatoid arthritis, Allograft rejection, Systemic lupus erythematosus, Inflammatory bowel disease, Asthma, Human T-cell lymphotropic virus type 1 infection, Th1 and Th2 cell differentiation, and Th17 cell differentiation), regulating metabolic processes (amino acid metabolism, lipid metabolism, and metabolism of cofactors and vitamins), cell growth and death, cellular community, signal transduction (including the cGMP-dependent protein kinase G signaling pathway and the TGF-β signaling pathway), and transport and catabolism processes (cell adhesion molecules signaling pathway and the phagosome signaling pathway) (Fig. 2D).

### NRF2-regulated key DEGs in PBMCs from patients with silicosis

**Fig. 3:**
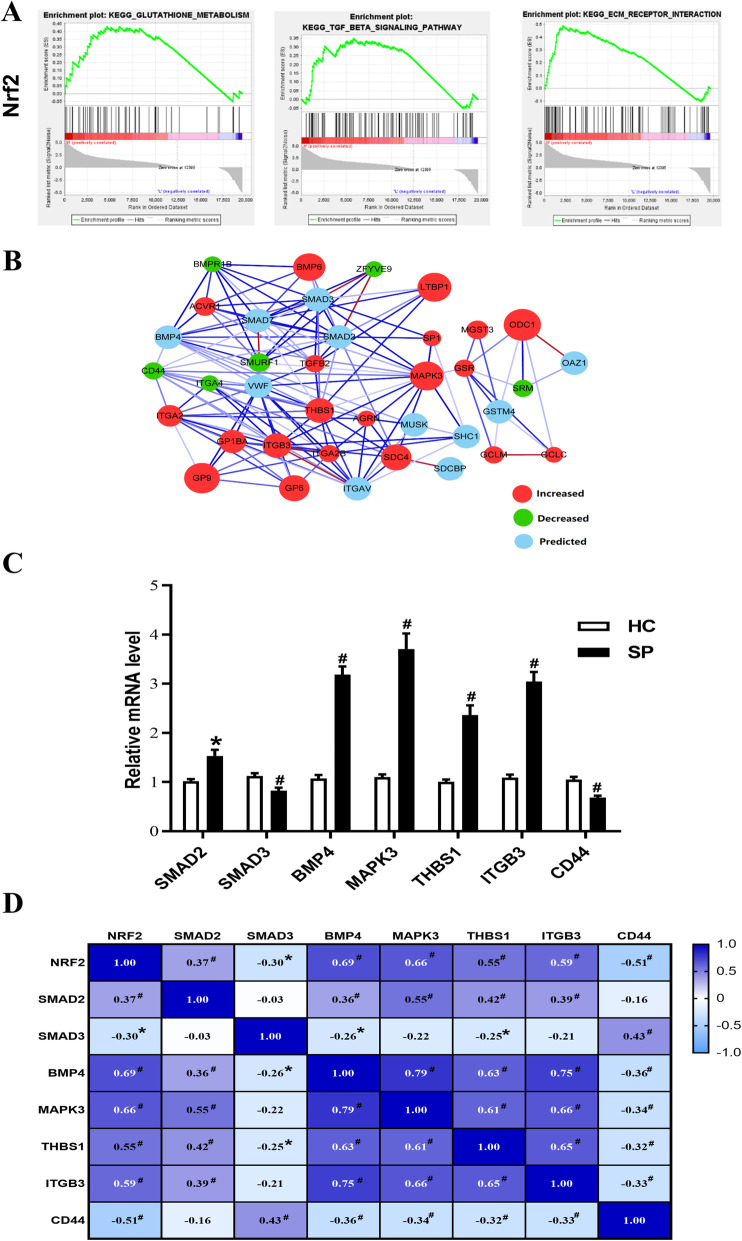
NRF2-regulated key DEGs in PBMCs from patients with silicosis. **A** Significant related genes and pathways in PBMCs of silicosis obtained by GSEA. ES, enrichment scores. Positive and negative ES indicate higher and lower expression in silicosis patients, respectively. **B** Protein-protein interaction (PPI) network of screened NRF2-related genes about glutathione metabolism, TGF-β signaling pathway and ECM receptor interaction were constructed a gene co-expression network. Red cycle nodes represent up-regulated genes, green cycle nodes represent down-regulated genes, and blue cycle nodes represent predicted genes. Each node represents one gene; edges indicate the interaction relationship. **C** NRF2-regulated DEGs associated with glutathione metabolism, TGF-β and ECM receptor interaction signaling pathway in silicosis patients PBMC. **D** Correlation of the mRNA expression levels among Nrf2, TGF-β pathway, and ECM pathway-related genes. **C** All data are expressed as fold change vs. HC, Error bar indicates mean ± SD (*n* = 36 per group). * *P* < 0.05 vs. HC, # *P* < 0.01 vs. HC

Based on the above results, NRF2 played a key role in silicosis. GSEA, a powerful tool to infer biological function, was performed and showed that genes associated with GSH metabolism, the TGF-β signaling pathway, and ECM receptor interaction signaling pathway, which were closely related to silicosis [40], were significantly enriched in the group of patients with silicosis (Fig. 3A). These observations suggested that NRF2 may be closely related to the lymphocyte oxidative stress state in patients with silicosis.

We screened 27 DEGs that genes associated with GSH metabolism, the TGF-β signaling pathway, and ECM receptor interaction signaling pathway from 1158 DEGs (Table 3). To further define the interaction between the screened 27 DEGs, the STRING database was used to construct a PPI network. The PPI network consisted of 37 nodes interacting with 144 edges (Fig. 3B). The PPI database was filtered using a combined score > 0.4 and the following top 10 hub genes were identified: *SMAD2* (degree = 15), *MAPK3* (degree = 15), *THBS1* (degree = 15), *SMAD3* (degree = 14), *ITGB3* (degree = 13), integrin alpha-V (*ITGAV*) (degree = 13), von Willebrand factor (*VWF*) (degree = 13), *BMP4* (degree = 13), *CD44* (degree = 12), and *SMAD7* (degree = 11).

**Table 3:** The screened 27 DEGs associated with lymphocyte oxidative stress state by GSEA analysis of NRF2. 27 key DEGs were identified from 1158 DEGs, which belong to the gene sets from GSH metabolism, the TGF-β signaling pathway, and ECM receptor interaction signaling pathway

Gene Symbol	Description	Log_2_FC	*t*	*P.*Value
GCLC	glutamate-cysteine ligase catalytic subunit	0.9761	3.5071	0.0130
THBS1	thrombospondin 1	1.8189	3.3611	0.0155
ITGA2B	integrin subunit alpha 2b	1.4937	7.8035	0.0003
BMP6	bone morphogenetic protein 6	1.4981	3.6512	0.0110
ZFYVE9	zinc finger FYVE-type containing 9	−1.2890	−2.8443	0.0299
ITGA2	integrin subunit alpha 2	1.1618	2.6027	0.0410
GP9	glycoprotein IX platelet	1.2380	4.6464	0.0036
ITGA4	integrin subunit alpha 4	−1.7863	−3.3991	0.0148
CD44	CD44 molecule	−1.2415	−4.3524	0.0050
SMURF1	SMAD specific E3 ubiquitin protein ligase 1	−0.6038	−2.5541	0.0438
TGFB2	transforming growth factor beta 2	1.5877	2.6729	0.0374
GSTM4	glutathione S-transferase mu 4	1.8561	2.4830	0.0482
GCLM	glutamate-cysteine ligase modifier subunit	0.6821	2.5854	0.0420
SDC4	syndecan 4	0.6770	2.7085	0.0357
AGRN	agrin	0.9977	2.8008	0.0316
MAPK3	mitogen-activated protein kinase 3	0.8754	2.5837	0.0421
ODC1	ornithine decarboxylase 1	0.7449	2.9195	0.0271
ACVR1	activin A receptor type 1	1.8224	2.9468	0.0261
GP6	glycoprotein VI platelet	1.2393	3.1317	0.0207
LTBP1	latent transforming growth factor beta binding protein 1	1.6414	4.7492	0.0033
BMPR1B	bone morphogenetic protein receptor type 1B	−2.4111	−2.7113	0.0355
GSR	glutathione-disulfide reductase	0.7830	2.6736	0.0374
SP1	SP1 transcription factor	1.1197	2.5086	0.0465
ITGB3	integrin subunit beta 3	1.1695	4.6047	0.0038
GP1BA	glycoprotein IB platelet alpha subunit	0.8702	2.7218	0.0351
MGST3	microsomal glutathione S-transferase 3	0.7892	2.9403	0.0264
SRM	spermidine synthase	−1.2838	−3.3023	0.0167

RT-qPCR was performed to determine the expression of NRF2-regulated DEGs in PBMCs from patients with silicosis, and confirm selected RNA-Seq findings. Steady-state mRNA levels of key genes associated with the TGF-β signaling pathway were determined. *SMAD2*, a downstream molecule of the TGF-β signaling pathway, was significantly higher in PBMCs from patients with silicosis. However, *SMAD3* mRNA levels were significantly lower than those in healthy controls. Next, it was confirmed that gene expression of *BMP4* and *MAPK3* were higher in PBMCs from patients with silicosis. Furthermore, the transcriptional factor related to cell-to-matrix interactions, *THBS1*, was significantly higher in PBMCs from patients with silicosis. Additionally, *ITGB3* was higher and *CD44* was significantly lower in PBMCs from patients with silicosis compared to health controls (Fig. 3C). The results of correlation analysis showed that the mRNA expression levels Nrf2 is significantly positively correlated with that of BMP4, MAPK3, and THGB3, is irrelevant with that of CD44 (Fig. 3D).

## Discussion

Nuclear factor erythroid 2-related factor 2 (NRF2) is a transcription activator that regulates the expression of target genes by binding antioxidant response elements (AREs) [41]. NRF2 is important for the coordinated upregulation of genes in response to oxidative stress. Silica causes the significant accumulation of ROS and activates the antioxidative protein NRF2 and its downstream proteins in the early stages of exposure to silica [42]. Immune cells with anti-inflammatory properties, especially monocytes/ macrophages are probably involved in the regulation of cellular immune on silicosis fibrosis [43–45]. The present study focused on the lymphocyte oxidative stress state and found that the number of monocytes and the expression of NRF2 and NRF2-dependent antioxidative genes was significantly increased in PBMCs from patients with silicosis. Additionally, it was confirmed that respiratory function was significantly lower in the patients with silicosis.

The respiratory dysfunction in patients with silicosis is closely related to the severity of pulmonary fibrosis [46]. The TGF-β pathway can play an important direct inducer role in the process of collagen transcription in the development of silicosis [47], as TGF-βs are overexpressed in fibrosis [48]. TGF-β signaling plays a key role in extracellular matrix remodeling, the epithelial-mesenchymal transition, and cell growth, migration, differentiation in fibrosis [49]. Generally, TGF-β signaling is modulated by the phosphorylation of the cytoplasmic SMAD signaling molecules, which results in their translocation to the nucleus [50]. TGF-β1 signaling molecules play a key role by promoting transdifferentiation the fibroblast into myofibroblasts, which promote collagen synthesis and ECM deposition in the pathology of silicosis [48].

The ECM, complex mixture of structural and functional macromolecules, has an important role in tissue fibrosis and in the maintenance of cell and tissue structure and function [51]. Integrins and other transmembrane molecules mediate specific interactions between cells and the ECM [52, 53]. These interactions have the direct and indirect effects of cellular activities, lead to adhesion, proliferation, apoptosis, migration, and differentiation. In addition, integrins act as ‘mechanoreceptors’ that they would provide a specific physical link between the cytoskeleton and the ECM [53].

In the present study, the differential expression of mRNAs in the silicosis was identified using RNA-Seq analysis. A total of 1158 dysregulated mRNAs were identified in PBMCs from patients with silicosis, including 475 upregulated and 683 downregulated mRNAs. GO analysis revealed that the functions of dysregulated mRNAs in PBMCs from patients with silicosis were related to the ECM, catalytic activity, oxidoreductase activity, transcription factor activity, metabolic processes, immune system processes, response to stimulus transcription, and biological adhesion. In KEGG pathway analysis, the dysregulated mRNAs were involved in regulating immune-related pathways, regulating metabolic processes, cellular community, cell adhesion molecules signaling pathway and the phagosome signaling pathway. Therefore, it is possible that the dysregulated mRNAs in these processes are involved in the pathogenesis of silicosis.

To confirm the results obtained by RNA-Seq analysis, seven differentially expressed mRNAs were selected to verify their expression in PBMCs from patients with silicosis using RT-qPCR. The results indicated that the expression of *SMAD2*, *MAPK3*, *THBS1*, *ITGB3*, and *BMP4* was increased, while the expression of *SMAD3* and *CD44* was significantly low expression in PBMCs from patients with silicosis, indicating consistent results with the RNA-Seq data. The TGF-β family of cytokines signals through receptor serine/threonine kinases to control cell behavior and fate [54]. These signals are propagated through the transcription factors SMAD2 and SMAD3 downstream of TGFβ. SMAD2 and SMAD3 belong to the SMAD protein family that mediate multiple signaling pathways as transcriptional modulators [55]. These findings indicate that dysregulation of the TGFβ1-SMAD signaling pathway may play an important role in the pathological process of silicosis, and are consistent with findings in animal experiments [56, 57]. SMAD proteins mediate signaling of TGF-β through its interaction with the SMAD anchor for receptor activation (SARA) protein [58]. In response to a TGF-β signal, SMAD proteins are phosphorylated by TGF-β receptors [59]. This study revealed that *BMP4* was a differential gene hub in PBMCs from patients with silicosis. *BMP4* encodes a secreted ligand of the TGF-β superfamily of proteins and activates SMAD family transcription factors that regulate gene expression [60]. These results showed that SMAD2 and SMAD3 had different trends in the two groups. Recently, SMAD2 and SMAD3 have shown different roles in the TGF-β signaling pathway during embryonic development [55]. The cell experiments revealed that BMP7 is associated with inhibiting silica-induced fibrosis through activated BMP7/SMAD and suppressed TGF-β/SMAD pathways [61, 62]. To date, BMP4 in the BMP protein family has not been reported in silicosis.

MAPK3 is especially involved in activation towards microtubule-associated protein-2 and the control of cell survival, proliferation and differentiation [63]. In the present study, *MAPK3* (*ERK1*) was significantly increased in PBMCs from patients with silicosis compared to that in the control group. Dysregulation of MAPK3 plays a significant role in the pathological processes of silicosis [16]. Crystal compounds in silicosis activate ROS, which activate the inflammasome through MAPK3 [16, 64]. In agreement with these findings, activation of MAPK3 (ERK1) and NF-κB in PBMCs is reported during oxidative stress [65].

The transcription factor thrombospondin-1 (*THBS1*) is an adhesive glycoprotein that mediates cell-to-cell and cell-to-matrix interactions. THBS1 binds to cell surface receptors, including fibrinogen, fibronectin, laminin, type V collagen and integrins, such as ITGB3 [66]. These studies suggest that THBS1 in human peripheral blood lymphocytes is involved in the regulation of pesticide-induced immune dysfunction [67].

The protein encoded by *CD44* is a cell-surface glycoprotein involved in cell-cell interactions, cell migration and adhesion [68]. It also interacts with other ligands, such as matrix metalloproteinases, and collagens [69]. CD44 blockade alleviates silica-induced fibrosis and improves pulmonary function in vivo [70].

These findings identify activation of the NRF2-mediated oxidative stress in PBMCs as a key contributor to the pathogenesis of patients with silicosis. Nonetheless, it has to be underlined that only the patients with silicosis stage I are included in this study and our results must be confirmed in larger trial. The other limitation of this study is that the present study remains unclear that changes specific to different cell types present in PBMCs. It will be necessary to study these patients further by establishing an in vitro model of PBMCs exposed to silica particles.

## Conclusions

Genome-wide mRNA profiling from PBMCs in patients with silicosis is identified. Overexpression of NRF2 and related signaling molecules are regulating the oxidative stress state in PBMCs from patients with silicosis. Therefore, NRF2 might serve as a novel preventive and therapeutic candidate for silicosis. The novel information provided by this study contribute to understand of the oxidative stress mechanism of silicosis.

## Data Availability

The datasets used and/or analyzed during the current study are available from the corresponding author on reasonable request.

## Abbreviations

AREs: Antioxidant response elements
BMI: Body mass index
BMP4: Bone morphogenetic protein 4
CAT: Catalase
DEGs: Differentially expressed genes
FEV1: Forced expiratory volume in one second
FVC: Forced vital capacity
GAPDH: Glyceraldehyde 3-phosphate dehydrogenase
GCLM: Glutamate-cysteine ligase modifier subunit
GO: Gene ontology
GPXs: Glutathione peroxidases
GR: Glutathione reductase
GSEA: Gene set enrichment analysis
GSH: Glutathione
ITBG3: Integrin beta-3
ITGAV: Integrin alpha-V
KEAP1: Kelch-like ECH-associated protein 1
KEGG: Kyoto Encyclopedia of Genes and Genomes
MAPK3: Mitogen-activated protein kinase 3
NK: Natural killer
NRF2: Nuclear factor erythroid 2-related factor 2
PBMCs: Peripheral blood mononuclear cells
PPI: Protein-protein interaction
RNA-Seq: RNA sequencing
ROS: Reactive oxygen species
SMAD3: Mothers against decapentaplegic homolog 3
SODs: Superoxide dismutases
STRING: Search Tool for the Retrieval of Interacting Genes/Proteins
THBS1: Thrombospondin-1
TGF: Transforming growth factor
vWF: von Willebrand factor

## References

[1] Riley L, Urbine D (2019). Chronic silicosis with progressive massive fibrosis. N Engl J Med.

[2] Leung C, Yu I, Chen W (2012). Silicosis. Lancet.

[3] The Lancet Respiratory Medicine (2019). The world is failing on silicosis. Lancet Respir Med.

[4] Han S, Chen H, Harvey MA, Stemn E, Cliff D. Focusing on coal workers’ lung diseases: a comparative analysis of China, Australia, and the United States. Int J Environ Res Public Health. 2018;15(11):2565. 10.3390/ijerph15112565. 10.3390/ijerph15112565 PMC6266950 30453500

[5] Steenland K, Goldsmith DF (1995). Silica exposure and autoimmune diseases. Am J Ind Med.

[6] Haustein UF (1998). Silica-induced lupus erythematosus. Acta Derm Venereol.

[7] Rosenman KD, Moore-Fuller M, Reilly MJ (1999). Connective tissue disease and silicosis. Am J Ind Med.

[8] Zhang XB (2013). Cellular reprogramming of human peripheral blood cells. Genomics Proteomics Bioinformatics.

[9] Murashima A, Takasaki Y, Ohgaki M, Hashimoto H, Shirai T, Hirose S (1990). Activated peripheral blood mononuclear cells detected by murine monoclonal antibodies to proliferating cell nuclear antigen in active lupus patients. J Clin Immunol.

[10] Hu S, Tao D, He P (2001). Immunophenotyping of lymphocyte T and B in the peripheral blood of systemic lupus erythematosus. J Tongji Med Univ.

[11] Otsuki T, Sakaguchi H, Tomokuni A, Aikoh T, Matsuki T, Isozaki Y, Hyodoh F, Kawakami Y, Kusaka M, Kita S, Ueki A (2000). Detection of alternatively spliced variant messages of Fas gene and mutational screening of Fas and Fas ligand coding regions in peripheral blood mononuclear cells derived from silicosis patients. Immunol Lett.

[12] Otsuki T, Sakaguchi H, Tomokuni A, Aikoh T, Matsuki T, Kawakami Y, Kusaka M, Ueki H, Kita S, Ueki A (1998). Soluble Fas mRNA is dominantly expressed in cases with silicosis. Immunology.

[13] Otsuki T, Tomokuni A, Sakaguchi H, Aikoh T, Matsuki T, Isozaki Y, Hyodoh F, Ueki H, Kusaka M, Kita S, Ueki A (2000). Over-expression of the decoy receptor 3 (DcR3) gene in peripheral blood mononuclear cells (PBMC) derived from silicosis patients. Clin Exp Immunol.

[14] Liu S, Hao C, Bao L, Zhao D, Zhang H, Hou J, Wang D, Chen H, Feng F, Yao W (2019). Silica particles mediate phenotypic and functional alteration of dendritic cells and induce Th2 cell polarization. Front Immunol.

[15] Joshi GN, Goetjen AM, Knecht DA (2015). Silica particles cause NADPH oxidase-independent ROS generation and transient phagolysosomal leakage. Mol Biol Cell.

[16] Harijith A, Ebenezer DL, Natarajan V (2014). Reactive oxygen species at the crossroads of inflammasome and inflammation. Front Physiol.

[17] Zhang ZQ, Zhang CZ, Shao B, Pang DH, Han GZ, Lin L (2019). Effects of abnormal expression of fusion and fission genes on the morphology and function of lung macrophage mitochondria in SiO-induced silicosis fibrosis in rats in vivo. Toxicol Lett.

[18] Zhang L, He YL, Li QZ, Hao XH, Zhang ZF, Yuan JX, Bai YP, Jin YL, Liu N, Chen G, Yun X, Yao SQ (2014). N-acetylcysteine alleviated silica-induced lung fibrosis in rats by down-regulation of ROS and mitochondrial apoptosis signaling. Toxicol Mech Methods.

[19] Huang H, Chen M, Liu F, Wu H, Wang J, Chen J, et al. N-acetylcysteine tiherapeutically protects against pulmonary fibrosis in a mouse model of silicosis. Biosci Rep. 2019;39(7):BSR20190681. 10.1042/BSR20190681. 10.1042/BSR20190681 PMC6639458 31273057

[20] Nakashima K, Sato T (2018). Regulatory role of heme oxygenase-1 in silica-induced lung injury. Respir Res.

[21] Yang J, Wang T, Li Y, Yao W, Ji X, Wu Q, Han L, Han R, Yan W, Yuan J, Ni C (2016). Earthworm extract attenuates silica-induced pulmonary fibrosis through Nrf2-dependent mechanisms. Lab Investig.

[22] Martinez FJ, de Andrade JA, Anstrom KJ, King TE, Raghu G (2014). Randomized trial of acetylcysteine in idiopathic pulmonary fibrosis. N Engl J Med.

[23] Moser MA, Chun OK. Vitamin C and heart health: a review based on findings from epidemiologic studies. Int J Mol Sci. 2016;17(8):1328. 10.3390/ijms17081328. 10.3390/ijms17081328 PMC5000725 27529239

[24] Castranova V (1994). Generation of oxygen radicals and mechanisms of injury prevention. Environ Health Perspect.

[25] Lazzarino G, Listorti I, Bilotta G, Capozzolo T, Amorini AM (2019). Water- and fat-soluble antioxidants in human seminal plasma and serum of fertile males. Antioxidants.

[26] Serrano I, Romero-Puertas MC, Sandalio LM, Olmedilla A (2015). The role of reactive oxygen species and nitric oxide in programmed cell death associated with self-incompatibility. J Exp Bot.

[27] Palabiyik SS, Girgin G, Tutkun E, Yilmaz OH, Baydar T (2013). Immunomodulation and oxidative stress in denim sandblasting workers: changes caused by silica exposure. Arh Hig Rada Toksikol.

[28] Marchitti SA, Chen Y, Thompson DC, Vasiliou V (2011). Ultraviolet radiation: cellular antioxidant response and the role of ocular aldehyde dehydrogenase enzymes. Eye Contact Lens.

[29] Yamamoto M, Kensler TW, Motohashi H (2018). The KEAP1-NRF2 system: a thiol-based sensor-effector apparatus for maintaining redox homeostasis. Physiol Rev.

[30] Re L, Martinez-Sanchez G, Bordicchia M, Malcangi G, Pocognoli A, Morales-Segura MA, Rothchild J, Rojas A (2014). Is ozone pre-conditioning effect linked to Nrf2/EpRE activation pathway in vivo? A preliminary result. Eur J Pharmacol.

[31] Kropat C, Mueller D, Boettler U, Zimmermann K, Heiss EH, Dirsch VM, Rogoll D, Melcher R, Richling E, Marko D (2013). Modulation of Nrf2-dependent gene transcription by bilberry anthocyanins in vivo. Mol Nutr Food Res.

[32] Zhao Y, Xu G, Li H, Chang M, Guan Y, Li Y, Wu W, Yao S (2020). Overexpression of endogenous lipoic acid synthase attenuates pulmonary fibrosis induced by crystalline silica in mice. Toxicol Lett.

[33] Ranu H, Wilde M, Madden B (2011). Pulmonary function tests. Ulster Med J.

[34] Miller MR, Crapo R, Hankinson J, Brusasco V, Burgos F, Casaburi R, Coates A, Enright P, van der Grinten CP, Gustafsson P, Jensen R, Johnson DC, MacIntyre N, McKay R, Navajas D, Pedersen OF, Pellegrino R, Viegi G, Wanger J (2005). General considerations for lung function testing. Eur Respir J.

[35] Consortium TGO (2017). Expansion of the gene ontology knowledgebase and resources. Nucleic Acids Res.

[36] Kanehisa M, Araki M, Goto S, Hattori M, Hirakawa M, Itoh M, Katayama T, Kawashima S, Okuda S, Tokimatsu T, Yamanishi Y (2008). KEGG for linking genomes to life and the environment. Nucleic Acids Res.

[37] Subramanian A, Tamayo P, Mootha VK, Mukherjee S, Ebert BL, Gillette MA, Paulovich A, Pomeroy SL, Golub TR, Lander ES, Mesirov JP (2005). Gene set enrichment analysis: a knowledge-based approach for interpreting genome-wide expression profiles. Proc Natl Acad Sci U S A.

[38] Bader GD, Hogue CW (2003). An automated method for finding molecular complexes in large protein interaction networks. BMC Bioinformatics.

[39] Azuaje FJ (2014). Selecting biologically informative genes in co-expression networks with a centrality score. Biol Direct.

[40] Abdelaziz R, Elkashef W, Said E (2016). Tadalafil reduces airway hyperactivity and protects against lung and respiratory airways dysfunction in a rat model of silicosis. Int Immunopharmacol.

[41] Lu Y, Sun Y, Liu Z, Lu Y, Zhu X, Lan B, et al. Activation of NRF2 ameliorates oxidative stress and cystogenesis in autosomal dominant polycystic kidney disease. Sci Transl Med. 2020;12(554):eaba3613. 10.1126/scitranslmed.aba3613. 10.1126/scitranslmed.aba3613 32727915

[42] Zhu Z, Yang G, Wang Y, Yang J, Gao A, Niu P, Tian L (2013). Suppression of thioredoxin system contributes to silica-induced oxidative stress and pulmonary fibrogenesis in rats. Toxicol Lett.

[43] Barbarin V, Arras M, Misson P, Delos M, McGarry B, Phan SH, Lison D, Huaux F (2004). Characterization of the effect of interleukin-10 on silica-induced lung fibrosis in mice. Am J Respir Cell Mol Biol.

[44] Nardi J, Nascimento S, Göethel G, Gauer B, Sauer E, Fão N, Cestonaro L, Peruzzi C, Souza J, Garcia SC (2018). Inflammatory and oxidative stress parameters as potential early biomarkers for silicosis. Clin Chim Acta.

[45] Liu H, Cheng Y, Yang J, Wang W, Fang S, Zhang W, Han B, Zhou Z, Yao H, Chao J, Liao H (2017). BBC3 in macrophages promoted pulmonary fibrosis development through inducing autophagy during silicosis. Cell Death Dis.

[46] Faria ACD, Carvalho ARS, Guimarães ARM, Lopes AJ, Melo PL (2019). Association of respiratory integer and fractional-order models with structural abnormalities in silicosis. Comput Methods Prog Biomed.

[47] Cai W, Xu H, Zhang B, Gao X, Li S, Wei Z, Li S, Mao N, Jin F, Li Y, Liu H, Yang F (2020). Differential expression of lncRNAs during silicosis and the role of LOC103691771 in myofibroblast differentiation induced by TGF-β1. Biomed Pharmacother.

[48] Yao W, Yang P, Qi Y, Jin L, Zhao A, Ding M, Wang D, Li Y, Hao C (2020). Transcriptome analysis reveals a protective role of liver X receptor alpha against silica particle-induced experimental silicosis. Sci Total Environ.

[49] Su J, Morgani SM, David CJ, Wang Q, Er EE, Huang YH, Basnet H, Zou Y, Shu W, Soni RK, Hendrickson RC, Hadjantonakis AK, Massagué J (2020). TGF-β orchestrates fibrogenic and developmental EMTs via the RAS effector RREB1. Nature.

[50] Li N, Feng F, Wu K, Zhang H, Zhang W, Wang W (2019). Inhibitory effects of astragaloside IV on silica-induced pulmonary fibrosis via inactivating TGF-β1/Smad3 signaling. Biomed Pharmacother.

[51] Mohan V, Das A, Sagi I (2020). Emerging roles of ECM remodeling processes in cancer. Semin Cancer Biol.

[52] Khadilkar RJ, Ho KYL, Venkatesh B, Tanentzapf G (2020). Integrins modulate extracellular matrix organization to control cell signaling during hematopoiesis. Curr Biol.

[53] Mitsou I, Multhaupt HAB, Couchman JR (2017). Proteoglycans, ion channels and cell-matrix adhesion. Biochem J.

[54] Chai Y, Ito Y, Han J (2003). TGF-beta signaling and its functional significance in regulating the fate of cranial neural crest cells. Crit Rev Oral Biol Med.

[55] Aragón E, Wang Q, Zou Y, Morgani SM, Ruiz L, Kaczmarska Z, Su J, Torner C, Tian L, Hu J, Shu W, Agrawal S, Gomes T, Márquez JA, Hadjantonakis AK, Macias MJ, Massagué J (2019). Structural basis for distinct roles of SMAD2 and SMAD3 in FOXH1 pioneer-directed TGF-β signaling. Genes Dev.

[56] Feng F, Li N, Cheng P, Zhang H, Wang H, Wang Y, Wang W (2020). Tanshinone IIA attenuates silica-induced pulmonary fibrosis via inhibition of TGF-β1-Smad signaling pathway. Biomed Pharmacother.

[57] Guo J, Yang Z, Jia Q, Bo C, Shao H, Zhang Z (2019). Pirfenidone inhibits epithelial-mesenchymal transition and pulmonary fibrosis in the rat silicosis model. Toxicol Lett.

[58] Bakkebø M, Huse K, Hilden VI, Forfang L, Myklebust JH, Smeland EB, Oksvold MP (2012). SARA is dispensable for functional TGF-β signaling. FEBS Lett.

[59] Ramachandran A, Vizán P, Das D, Chakravarty P, Vogt J, Rogers KW (2018). TGF-β uses a novel mode of receptor activation to phosphorylate SMAD1/5 and induce epithelial-to-mesenchymal transition. Elife.

[60] Takebe Y, Tsujigiwa H, Katase N, Siar CH, Takabatake K, Fujii M, Tamamura R, Nakano K, Nagatsuka H (2017). Parenchyma-stromal interactions induce fibrosis by secreting CCN2 and promote osteoclastogenesis by stimulating RANKL and CD68 through activated TGF-β/BMP4 in ameloblastoma. J Oral Pathol Med.

[61] Liang D, Wang Y, Zhu Z, Yang G, An G, Li X, Niu P, Chen L, Tian L (2016). BMP-7 attenuated silica-induced pulmonary fibrosis through modulation of the balance between TGF-β/Smad and BMP-7/Smad signaling pathway. Chem Biol Interact.

[62] Chen M, Wan B, Zhu S, Zhang F, Jin J, Li X, Wang X, Lv Y, Chen C, Lv T, Song Y (2019). Geranylgeranyl diphosphate synthase deficiency aggravates lung fibrosis in mice by modulating TGF-β1/BMP-4 signaling. Biol Chem.

[63] Maitra S, Das D, Ghosh P, Hajra S, Roy SS, Bhattacharya S (2014). High cAMP attenuation of insulin-stimulated meiotic G2-M1 transition in zebrafish oocytes: interaction between the cAMP-dependent protein kinase (PKA) and the MAPK3/1 pathways. Mol Cell Endocrinol.

[64] Chu L, Wang T, Hu Y, Gu Y, Su Z, Jiang H (2013). Activation of Egr-1 in human lung epithelial cells exposed to silica through MAPKs signaling pathways. PLoS One.

[65] Akhter N, Madhoun A, Arefanian H, Wilson A, Kochumon S, Thomas R, Shenouda S, Al-Mulla F, Ahmad R, Sindhu S (2019). Oxidative stress induces expression of the toll-like receptors (TLRs) 2 and 4 in the human peripheral blood mononuclear cells: implications for metabolic inflammation. Cell Physiol Biochem.

[66] Calzada MJ, Roberts DD. Novel integrin antagonists derived from thrombospondins. Curr Pharm Des. 2005;11(7):849-66. 10.2174/1381612053381792. 10.2174/1381612053381792 15777239

[67] Mandarapu R, Prakhya BM (2016). Exposure to cypermethrin and mancozeb alters the expression profile of THBS1, SPP1, FEZ1 and GPNMB in human peripheral blood mononuclear cells. J Immunotoxicol.

[68] Chen C, Zhao S, Karnad A, Freeman JW (2018). The biology and role of CD44 in cancer progression: therapeutic implications. J Hematol Oncol.

[69] Pongcharoen P, Jinawath A, Tohtong R (2011). Silencing of CD44 by siRNA suppressed invasion, migration and adhesion to matrix, but not secretion of MMPs, of cholangiocarcinoma cells. Clin Exp Metastasis.

[70] Li S, Li C, Zhang Y, He X, Chen X, Zeng X, Liu F, Chen Y, Chen J (2019). Targeting mechanics-induced fibroblast activation through CD44-RhoA-YAP pathway ameliorates crystalline silica-induced silicosis. Theranostics.

